# Type-specific molecular signaling architectures and synaptic plasticity of *Drosophila* olfactory sensory neurons

**DOI:** 10.3389/fncel.2025.1579821

**Published:** 2025-08-26

**Authors:** Namrata Acharya, Eric Wiesel, Mareike Selcho, Nadine Ehmann, Marius Lamberty, Bill S. Hansson, Dieter Wicher, Robert J. Kittel

**Affiliations:** ^1^Department of Animal Physiology, Faculty of Life Sciences, Leipzig University, Leipzig, Germany; ^2^Department of Evolutionary Neuroethology, Max Planck Institute for Chemical Ecology, Jena, Germany

**Keywords:** active zone, dendrite, homeostatic synaptic plasticity, geosmin, olfaction

## Abstract

Olfactory sensory neurons (OSNs) detect odours at a wide range of intensities. In *Drosophila*, volatile compounds bind to specific odorant receptors (ORs), which tune the sensitivity of chemoreception. To test whether additional mechanisms underlie odour-specific neuronal processing, we analysed the spatial distribution of ORs in dendrites and investigated OSN synapses in the antennal lobe, the first relay station of the olfactory pathway. Here, we studied the molecular structure and plasticity of the presynaptic active zone (AZ), the specialized site of neurotransmitter release. We focused on a highly sensitive OSN type that expresses the receptor Or56a and is exclusively activated by geosmin, an odorant signalling ecologically harmful microorganisms. Our results uncover a differential arrangement of dendritic ORs and core AZ proteins in alarm odour-detecting Or56a compared to conventional food-odour detecting OSNs. Interestingly, the data also show that Or56a OSNs display a limited capacity for homeostatic plasticity in response to a genetic reduction of presynaptic release probability. We hypothesise that this feature reflects the basal tuning of geosmin-sensing neurons towards maximum levels of performance.

## Introduction

An organism must make keen use of its senses to navigate complex environments effectively. For many airborne insects, olfaction is essential for locating food sources, finding mates, identifying suitable breeding substrates, and avoiding predators and other dangers (Hansson and Stensmyr, 2011). To tackle these challenges, insects have evolved a sophisticated olfactory system capable of detecting airborne odour plumes over considerable distances (Koehl, 2006; Missbach et al., 2014; Van Breugel and Dickinson, 2014; Wicher, 2018).

In *Drosophila melanogaster*, chemoreception occurs across various organs, but the primary olfactory organ is located in the third segment of the antenna. This segment, known as the funiculus, contains hair-like structures called sensilla which house approximately 50 different types of olfactory sensory neurons (OSNs), collectively forming the olfactory arsenal of *Drosophila melanogaster* (De Bruyne et al., 2001; Laissue and Vosshall, 2008; Grabe et al., 2016; Benton, 2022). The OSNs are categorized based on their expression of specific odorant receptors (ORs) or ionotropic receptors (IRs). Depending on the expressed receptor, they are activated by different compounds, but also differ in their sensitivity, cell morphology, electrical activity and tuning abilities (Fishilevich and Vosshall, 2005; Hallem and Carlson, 2006; Mukunda et al., 2016; Gonzales et al., 2021; Jain et al., 2021; Wiesel et al., 2022; Halty-deLeon et al., 2024). In the following, we focus on ORs.

All OSN types consist of three segments. The outermost segment, the dendrite, is freely accessible and is surrounded by a sensillum lymph maintained by support cells (Seidl, 1992; Prelic et al., 2022). Here odour molecules can bind to ORs and initiate a neuronal response (Kaupp, 2010). The OR complex is composed of specific ligand-binding subunits (OrX) and the broadly-expressed co-receptor protein (Orco). Together they form non-selective cation channels passing Na^+^, K^+^, and Ca^2+^ (Larsson et al., 2004; Sato et al., 2008; Wicher et al., 2008). ORs need to be able to detect odours in a wide range of concentrations, from faint filaments at larger distance from the source to high concentrations and permanent presence near the source (Wicher, 2018). To achieve this, OSNs have developed mechanisms to tune their performance dynamically according to changing physiological requirements (Getahun et al., 2016). Sensory systems are challenged to optimize the degree of resolution (Młynarski and Hermundstad, 2018). They must manoeuvre between metabolically expensive highest resolution and less costly lower resolved representations of the environment, which may cause interpretational errors and lead to inappropriate behavioural responses. In addition to regulatory processes such as desensitization (Poudel et al., 2021), a unique property of OSNs detecting food odours is their capability to sensitize upon repeated stimulation with highly diluted odours below the detection threshold (Getahun et al., 2013). Mechanisms contributing to sensitization include Ca^2+^ influx into the sensory neurons, OR protein phosphorylation and calmodulin action on ORs (Sargsyan et al., 2011; Mukunda et al., 2014, 2016).

Following odour detection in the outer dendrite, information is carried as action potentials along axons to specific glomeruli of the antennal lobe [AL; (Vosshall and Stocker, 2007; Grabe et al., 2016)], where OSNs form cholinergic synapses with partner neurons. At chemical synapses, neurotransmitter substances are released from synaptic vesicles upon Ca^2+^ influx at the highly specialized presynaptic active zone (AZ). Here, complex protein interactions give rise to the speed and precision of neurotransmission (Südhof, 2012). Importantly, AZs display considerable heterogeneities at molecular, functional and ultrastructural levels, not only between different species and cell types, but also between individual AZs of the same neuron (Atwood and Karunanithi, 2002; Melom et al., 2013; Ehmann et al., 2014; Peled et al., 2014; Reddy-Alla et al., 2017). Moreover, AZ properties can be modified in an activity-dependent manner on timescales ranging from milliseconds to days (Regehr, 2012; Monday et al., 2018). The plasticity and diversity of AZs are important for information processing by the nervous system but the underlying cellular and molecular mechanisms are not well understood.

The Or56a receptor is of great ecological importance for *Drosophila melanogaster* by exclusively detecting geosmin, an odour released by toxin-producing microbes (Stensmyr et al., 2012). The sensitivity of the OSNs expressing Or56a is extraordinarily high (Halty-deLeon et al., 2024) and comparable to moth pheromone receptors that can detect single odorant molecules (Stengl, 2010). Or56a neurons target exclusively the DA2 glomerulus in the antennal lobe and activation of DA2 elicits aversive behaviour overriding input from other olfactory pathways (Stensmyr et al., 2012). When Or56a is heterologously expressed, repetitive stimulation with a near-threshold concentration of a synthetic OR agonist can lead to sensitization of the receptor (Mukunda et al., 2014). However, native Or56a-expressing OSNs do not exhibit sensitization (Halty-deLeon et al., 2024) in contrast to the tuneable food odour-detecting OSNs expressing, e.g., Or22a. Unlike Or22a expressing OSNs, which display a moderate sensitivity to odours under resting conditions, the Or56a neurons seem to be set to their highest sensitivity by default.

In the present study we set out to investigate whether specific molecular and ultrastructural layouts of OSN sub-compartments correlate with neuronal performance features. By comparing the dendrites and presynaptic AZs of Or22a and Or56a OSNs, we identify a differential localization of proteins involved in signal reception and signal transmission and demonstrate that these two OSN subtypes have distinct properties of synaptic plasticity.

## Materials and methods

### Fly stocks

Flies were raised on standard cornmeal and molasses medium at 25°C except for the RNAi experiments where all genotypes were raised at 29°C. The following fly strains were used: *or22a-GAL4* (BDSC 9951), *or56a-GAL4* (BDSC 23896), *20XUAS-IVS-mCD8::GFP* (BDSC 32194), *UAS-cac-RNAi* [VDRC GD 5551; (Dietzl et al., 2007; Rozenfeld et al., 2023)], *10XUAS-myr::GFP, UAS-brp[D3]::mRFP/TM6B, Tb* (Fouquet et al., 2009), *UAS-GCamp6f; or22a-GAL4* (Halty-deLeon et al., 2024), *UAS-GCamp6f; or56a-GAL4/TM6B* (Halty-deLeon et al., 2024), *UAS-N-GFP-orco; or22a-GAL4, orco^1^* (Jain et al., 2023), *UAS-N-GFP-orco; or56a-GAL4, orco^1^* (Jain et al., 2023).

### Confocal microscopy

Female flies, 5–8 days of age (0 or 5 days for homeostasis experiments) were dissected on ice and brains were fixed in 4% paraformaldehyde (PFA) for 2 h (or 20 min in methanol for Unc13A) at room temperature. The samples were then washed 6 × 10 min with 0.3% PBT (PBS with 0.3% Triton X-100, Sigma Aldrich) and blocked for 1 h or overnight with 5% normal goat serum (NGS) in PBT. Following incubation with primary antibodies for 24 h (homeostasis experiments) or 48 h the samples were washed 6 × 10 min with 0.3% PBT and then incubated with secondary antibodies for 24 h. After final washing steps 6 × 10 min with 0.3% PBT and 1 × 10 min with PBS the samples were mounted in Vectashield (Vector Laboratories) and stored at 4°C. The following antibodies were used: mouse-*α*-Brp [nc82, 1:10, DSHB, AB_2314866; (Wagh et al., 2006)], guinea pig-α-Unc13A [1:100; (Böhme et al., 2016)], rabbit-α-Unc13B [1:300; (Böhme et al., 2016)], rabbit-α-Syd-1 [1:250; (Owald et al., 2010)], rabbit-α-GFP (1:400, Thermo Fisher Scientific, AB_2536526), mouse-α-GFP (1:500, Sigma-Aldrich, AB_2827519), rat-α-mCherry (1:1000, Thermo Fisher Scientific, AB_2536611), goat-α-mouse-StarRed (1:200, Abberior, AB_3068620), goat-α-mouse-AF488 (1:200, Thermo Fisher Scientific, AB_2534069), goat-α-rabbit-AF488 (1:200, Thermo Fisher Scientific, AB_2576217), goat-α-rat-Cy3 (1:250, Thermo Fisher Scientific, AB_2534031), goat-α-guinea pig-StarRed (1:200, Abberior, AB_306823). Image stacks of whole-mount brains were acquired with an upright STED microscope in confocal mode (Infinity Line, Abberior Instruments), 60x/1.42 NA oil immersion objective, 0.5 μm stack size (*α*-Brp, α-Unc13A, α-Unc13B, and α-Syd1) or with a Zeiss LSM 800 microscope (Carl Zeiss Microscopy), 63x/1.4 NA oil immersion objective, 1 μm stack size (Brp^short^:mRFP). Identical laser settings were used for all genotypes in each imaging session. Image analysis was carried out with ImageJ (National Institutes of Health) on each image of the stack. The GFP signals were used to generate glomerulus-specific masks, which were overlaid with the Brp, Unc13A, Unc13B or Syd-1 channels. Individual puncta were detected with the “Find Maxima” command and quantified via “Analyze Particles.” The results for Brp homeostasis at day 5 were replicated in an independent experiment (significantly more AZs following Cac knockdown in Or22a and no difference in Or56a OSNs).

### STED microscopy

Dissected fly antennae were mounted in OCT compound (VWR Chemicals) and frozen for 20 min. A cryostat was used to cut 12 μm sections collected on SuperFrost Plus microscope slides (Epredia). Immediately after sectioning, the samples were fixed for 10 min in 2% PFA and washed 2 × 10 min in PBS. The slides were then transferred to a humidified chamber and blocked for 30 min with 2% NGS in PBS. The samples were incubated with the primary antibody in blocking solution at 4°C overnight. The following day, the sections were washed 4 × 10 min in PBS and blocked for 30 min before incubating with the secondary antibody for 2 h at 25°C. After final washing steps 4 × 5 min in PBS and 1 × 5 min in distilled water, the sections were mounted in Vectashield (Vector Laboratories) and stored at 4°C. The following antibodies were used: mouse-α-GFP (1:200, Thermo Fisher Scientific, A-11120) and goat-α-mouse-StarRed (1:100, Abberior, STRED-1001-500UG). Images were acquired with an upright STED microscope (Infinity Line, Abberior Instruments), equipped with an 60x/1.42 NA oil immersion objective, and identical laser settings for all experiments. The area occupied by Orco^GFP^ was measured manually in ImageJ (National Institutes of Health).

### Electron microscopy data

Codex (FlyWire Brain Dataset FAFB v783; http://dx.doi.org/10.13140/RG.2.2.35928.67844) and FlyWire Neuroglancer were used to identify the OSNs (Zheng et al., 2018; Dorkenwald et al., 2022, 2024; Schlegel et al., 2024). OSN AZs within the DA2 and DM2 glomeruli of the right antennal lobe were counted manually using FlyWire Neuroglancer. AZs were identified by electron dense membranes, vesicle clusters, and adjacent dendritic arborizations. Each T-bar-like filamentous structure was counted as one AZ, even when multiple T-bars shared one electron dense presynaptic membrane.

### Statistics

Data were analysed with Prism 9 (GraphPad). Group means were compared with an unpaired t-test, unless the assumption of normal sample distribution was violated according to the Shapiro–Wilk test. In this case, a non-parametric Mann–Whitney test was employed.

## Results

**Figure 1:**
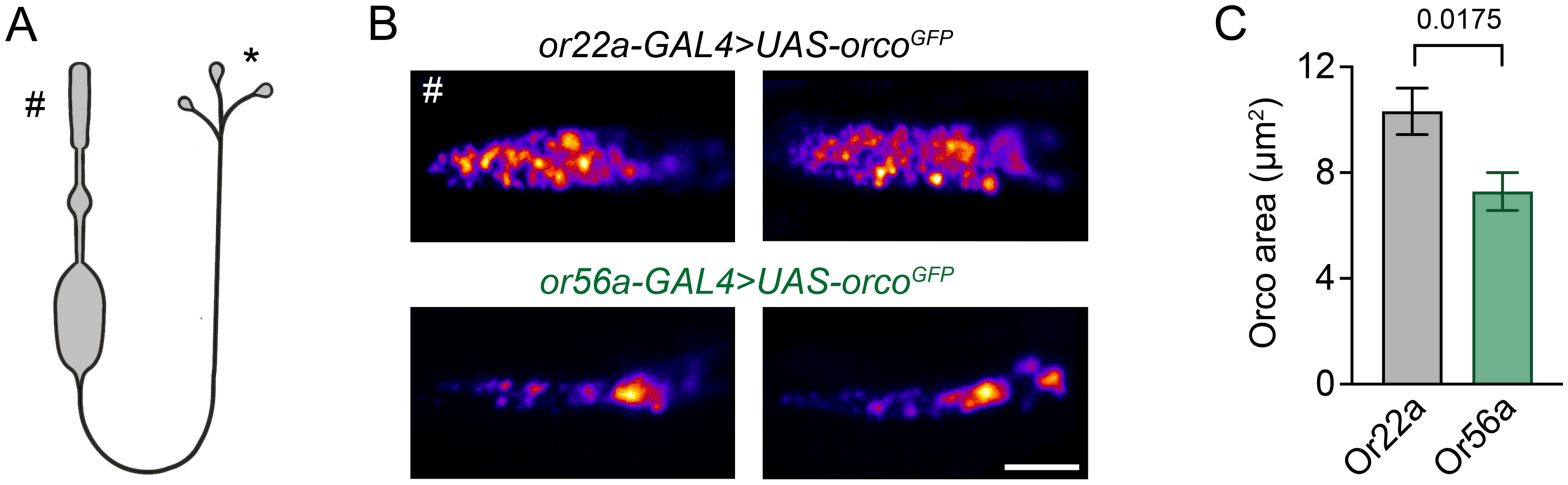
Dendritic arrangement of ORs. **(A)** Schematic depiction of an OSN (not to scale), # outer dendrite in the periphery, * presynapses in the AL. **(B)** Example STED images of GFP-tagged Orco in cryosections of outer dendrites of food odour (*or22a-GAL4 > UAS-orco^GFP^*) and alarm odour (*or56a-GAL4 > UAS-orco^GFP^*) detecting OSNs. **(C)** Orco is distributed over a smaller area in Or56a OSNs (Or22a: 10.3 ± 0.9 μm^2^, *n* = 25; Or56a: 7.3 ± 0.7 μm^2^ SEM, *n* = 22 OSNs; *p* = 0.0175 Mann–Whitney test). Scale bar 2 μm, data are presented as mean ± SEM.

Given the different sensitivities of food odour-detecting and alarm odour-detecting OSNs, we first asked how the receptors are distributed within the outer segments of the dendrites. To this end, we used the high spatial resolution of Stimulated Emission Depletion Microscopy [STED; (Hell, 2007)] to measure the arrangement of the Orco co-receptor tagged with GFP and expressed in the *orco* null mutant background (Jain et al., 2023) in Or22a and Or56a expressing neurons. Interestingly, ORs occupied a smaller area in Or56a OSNs (Figure 1), which is in line with their smaller dendritic size compared to Or22a-expressing OSNs (Gonzales et al., 2021; Halty-deLeon et al., 2024). Thus, regarding receptor arrangement, we observed no obvious features that would explain the different sensitivities of the respective OSNs.

**Figure 2:**
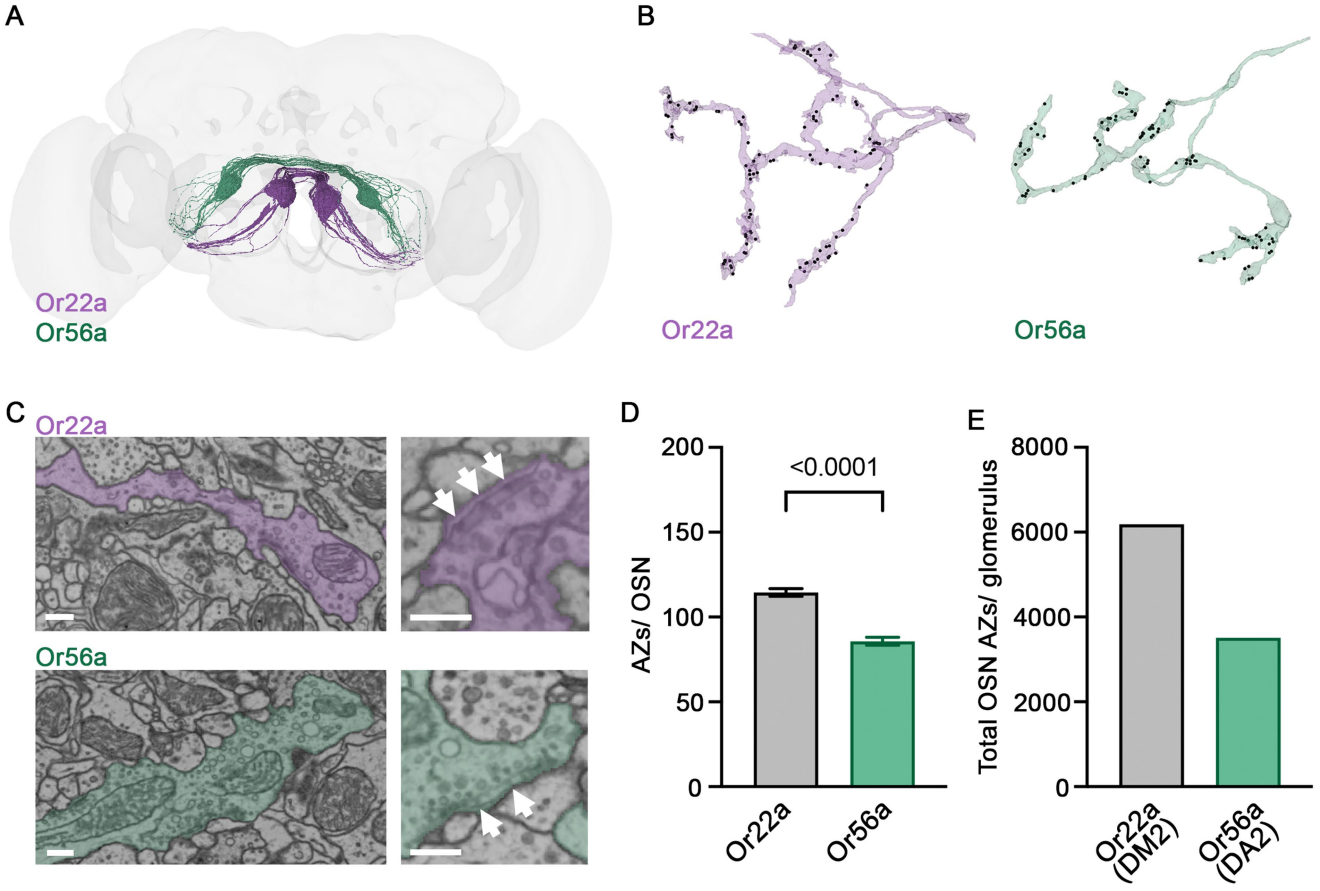
Presynaptic ultrastructure of OSNs. **(A)** 54 Or22a OSNs (magenta) and 41 Or56a OSNs (green) reconstructed with FlyWire. **(B)** Example arborisations of an Or22a OSN and an Or56a OSN within the DM2 and DA2 glomeruli, respectively. AZs labelled as black dots. **(C)** Ultrastructure of presynaptic sites within the respective glomerulus. Presynaptic T-bars (arrows) lie close to each other. **(D)** Quantification of AZs per OSN within the DM2 (Or22a) or DA2 (Or56a) glomerulus (Or22a: 115 ± 2 SEM *n* = 54, Or56a: 86 ± 2 SEM *n* = 41 OSNs; *p* < 0.0001 unpaired t-test). **(E)** Total OSN AZ number within the DM2 (Or22a) and DA2 (Or56a) glomerulus of the right antennal lobe [Or22a, DM2 glomerulus: 6183 AZs (54 cells); Or56a, DA2 glomerulus: 3513 AZs (41 cells)]. Scale bars 300 nm, data are presented as mean ± SEM.

Next, we shifted our focus from signal reception in the OSN dendrite to the site of signal transmission at the presynaptic AZ. We made use of the recently published *Drosophila* connectome made accessible via FlyWire Neuroglancer and Codex (Zheng et al., 2018; Dorkenwald et al., 2022, 2024) and compared the ultrastructure of presynaptic sites of Or56a and Or22a neurons in DA2 and DM2 glomeruli, respectively (Figure 2A). A total of 40 Or56a OSNs and 54 Or22a OSNs have been annotated in the FlyWire Brain Dataset (FAFB v783). We identified an additional afferent neuron in the DA2 glomeruli, thus increasing the total number of Or56a OSNs to 41. The smaller number of OSNs innervating the DA2 glomerulus is consistent with its smaller volume compared to the DM2 glomerulus (Grabe et al., 2016). Subsequently, we counted OSN AZs within the DA2 and DM2 glomeruli of the right antennal lobe (Figure 2B). The DA2 glomerulus is innervated by 22 ipsilateral and 19 contralateral Or56a OSNs, while the DM2 glomerulus receives input from 25 ipsilateral and 29 contralateral Or22a OSNs (Figure 2A). The AZ number per neuron was smaller in Or56a than in Or22a OSNs (Figure 2D). Consistent with the above results, the total number of AZs per glomerulus was also smaller for Or56a compared to Or22a OSNs (Figure 2E).

**Figure 3:**
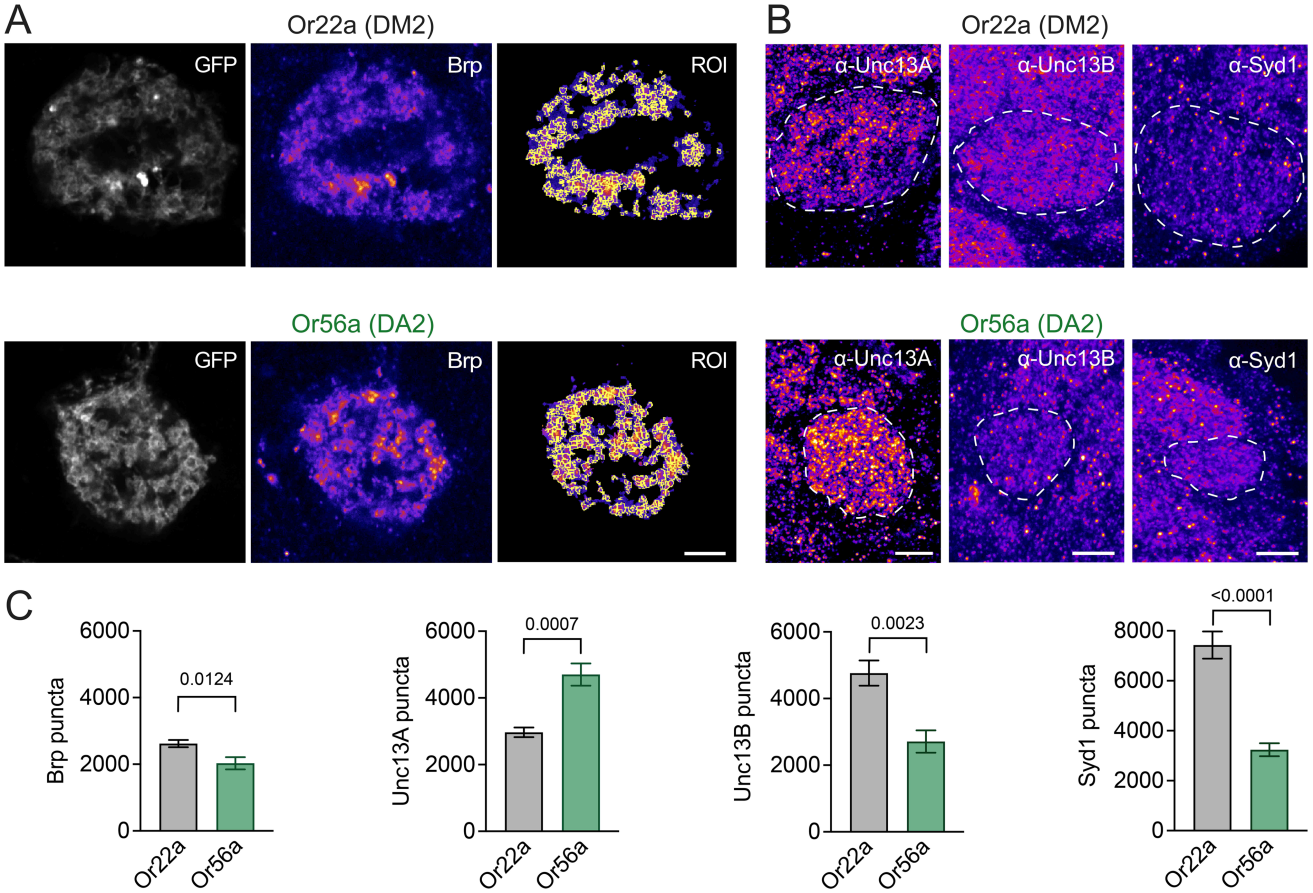
OSN AZ proteins. **(A)** Example images of Brp^short^::mRFP illustrating spot detection in regions of interest (ROI) within GFP-labelled DM2 (*or22a-GAL4 > UAS-brp^short^::mRFP, UAS-myr::GFP*) or DA2 (*or56a-GAL4 > UAS-brp^short^::mRFP, UAS-myr::GFP*) glomeruli. **(B)** Example images (maximal projections of 5 confocal stacks) of Unc13A, Unc13B, and Syd-1 antibody stainings in DM2 (*or22a-GAL4 > UAS-mcd8::GFP* or *or22a-GAL4 > UAS-gcamp6f*) and DA2 (*or56a-GAL4 > UAS-mcd8::GFP* or *or56a-GAL4 > UAS-gcamp6f*) glomeruli (GFP-defined glomeruli indicated by dashed line). **(C)** Quantification of Brp (Or22a: 2620 ± 108, *n* = 10; Or56a: 2029 ± 183 SEM, *n* = 10 glomeruli; *p* = 0.0124 unpaired *t*-test), Unc13A (Or22a: 2968 ± 146, *n* = 6; Or56a: 4701 ± 332 SEM, *n* = 6 glomeruli; *p* = 0.0007 unpaired *t*-test), Unc13B (Or22a: 4763 ± 378, *n* = 6; Or56a: 2710 ± 335 SEM, *n* = 6 glomeruli; *p* = 0.0023 unpaired *t*-test), and Syd-1 puncta (Or22a: 7430 ± 541, *n* = 6; Or56a: 3242 ± 260 SEM, *n* = 6 glomeruli; *p* < 0.0001 unpaired *t*-test). Scale bars 5 μm, data are presented as mean ± SEM.

Following these ultrastructural findings, we asked whether Or56a and Or22a OSNs also display differences in the molecular composition of neurotransmitter release sites. The ELKS/Cast family member Bruchpilot (Brp) is a major structural component of the AZ cytomatrix, which appears as T-bars in electron micrographs [Figure 2C; (Kittel et al., 2006; Wagh et al., 2006)]. Transgenic expression of a photoprotein-tagged Brp fragment (Brp^short^::mRFP) reliably reports endogenous AZ Brp levels without disrupting the presynaptic organisation (Fouquet et al., 2009; Kremer et al., 2010). In line with the EM data, confocal fluorescence images showed a smaller number of Brp-positive AZs in Or56a (DA2 glomerulus) than in Or22a (DM2 glomerulus) OSNs (Figures 3A,C). As expected, given the higher spatial resolution of the EM reconstructions, the total number of AZs was smaller in the confocal image stacks. Next, we imaged the release site marker Unc13A. Strikingly, Unc13A counts were strongly increased in DA2 glomeruli, innervated by Or56a OSNs (Figure 3B). This observation is particularly interesting given that high Unc13A levels correlate with a high neurotransmitter release probability at AZs (Böhme et al., 2016; Fulterer et al., 2018). Conversely, the AZ proteins Unc13B and Syd-1, which are both connected to low release probability were less abundant in Or56a-expressing OSNs (Figure 3C).

**Figure 4:**
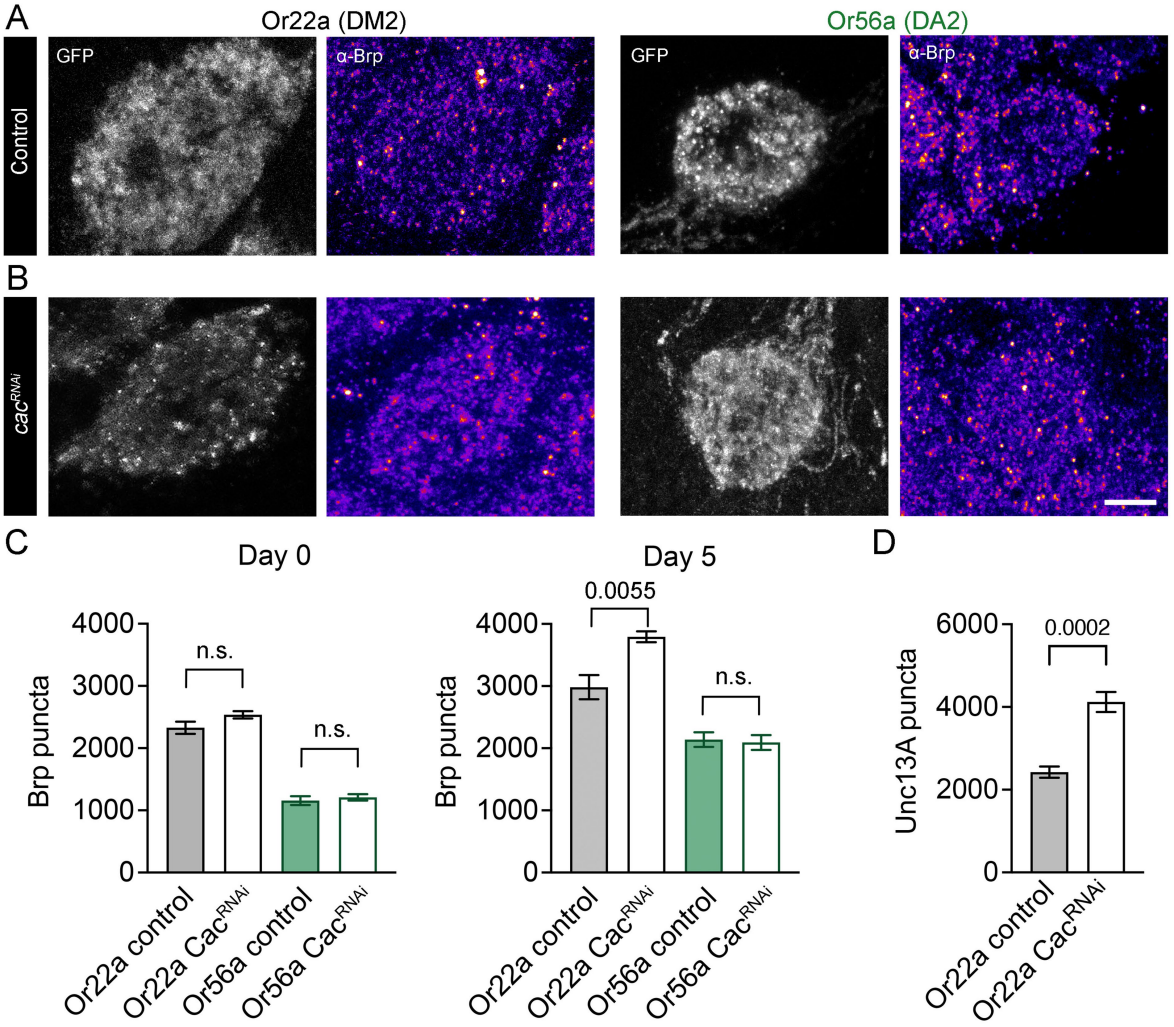
Homeostatic synaptic plasticity of OSNs. **(A)** Antibody stainings against Brp (monoclonal antibody nc82) in DM2 and DA2 glomeruli of 5-day-old controls (DM2: *or22a-GAL4 > UAS-gcamp6f*; DA2: *or56a-GAL4 > UAS-gcamp6f*) and **(B)** Cac knockdown flies (DM2: *or22a-GAL4 > UAS-cac^RNAi^, UAS-gcamp6f*; DA2: *or56a-GAL4 > UAS-cac^RNAi^, UAS-gcamp6f*). Shown are maximal projections of 9 confocal stacks. **(C)** Quantification of Brp levels in newly eclosed (day 0; Or22a control: 2328 ± 98, *n* = 8, Or22a Cac^RNAi^: 2537 ± 59 SEM, *n* = 8 glomeruli, *p* = 0.0893 unpaired *t*-test; Or56a control: 1156 ± 71, *n* = 8, Or56a Cac^RNAi^: 1210 ± 51 SEM, *n* = 8 glomeruli, *p* = 0.5521 unpaired *t*-test) and 5-day-old flies (day 5; Or22a control: 2983 ± 196, *n* = 8, Or22a Cac^RNAi^: 3794 ± 87 SEM, *n* = 6 glomeruli, *p* = 0.0055 unpaired t-test; Or56a control: 2139 ± 117, *n* = 8, Or56a Cac^RNAi^: 2093 ± 119 SEM, *n* = 8 glomeruli, *p* = 0.7854 unpaired *t*-test). **(D)** Antibody stainings show an increase in Unc13A levels upon Cac knockdown in Or22a-expressing OSNs (*or22a-GAL4 > UAS-gcamp6f*: 2427 ± 136, *n* = 6, *or22a-GAL4 > UAS-cac^RNAi^, UAS-gcamp6f*: 4121 ± 242, *n* = 12 glomeruli, *p* = 0.0002 unpaired *t*-test, 5-7-day-old flies). Scale bar 5 μm, n.s.: no significant difference, data are presented as mean ± SEM.

Recent work demonstrated that OSNs can undergo homeostatic synaptic plasticity to compensate for a drop in presynaptic release probability. When the AZ calcium channel subunit Cacophony [Cac; (Kawasaki et al., 2004)] is knocked down in all Orco-expressing neurons via RNAi, OSNs increase the number of Brp-positive AZs onto projection neurons following eclosion, thereby maintaining reliable neural coding and odour-driven behaviour (Rozenfeld et al., 2023). However, it is not known whether all OSN types have a similar capacity for homeostatic plasticity. Unlike food odour-detecting OSNs, Or56a-expressing neurons appear non-tuneable at the level of odour reception in dendritic and somatic compartments (Halty-deLeon et al., 2024). We therefore examined whether Or56a OSNs are also characterized by differences in presynaptic plasticity. As reported for a broad population of OSNs (Rozenfeld et al., 2023), Or22a neurons with reduced Cac levels had a normal AZ count shortly after eclosion (Figures 4A,C) but compensated for Cac knockdown by increasing AZ numbers within several days, reflected by an increase in the number of both Brp and Unc13A puncta (Figures 4B–D). In contrast, Or56a neurons showed no such homeostatic compensation through AZ addition (Figures 4B,C), matching their lack of peripheral plasticity.

## Discussion

OSNs detecting the alarm odour geosmin are two orders of magnitude more sensitive than the food odour-detecting OSNs expressing Or22a (Halty-deLeon et al., 2024). The distribution of Or56a over a smaller area (Figure 1C) may imply a higher density of ORs, though this could not be resolved with STED microscopy. If this were the case, such a tightly packed arrangement could perhaps enable cooperativity as observed for bacterial chemoreceptors (Hazelbauer et al., 2008). *E. coli* chemoreceptors, for example, form arrays that allow high amplification of chemosignals. With densely packed receptors, one odour molecule may activate more than one receptor (Kaizu et al., 2014) and in the case of Or56a, which also passes Ca^2+^ ions, such an effect could lead to stronger Ca^2+^ signals. Or56a expressing OSNs possess small inner dendrites equipped with only a few mitochondria (Halty-deLeon et al., 2024). These OSNs can hardly buffer Ca^2+^ signals in their inner dendrites and the signals can propagate through the neuron without attenuation and initiate action potentials (Berridge, 1998). Such effects may in sum help to explain the high sensitivity of Or56a-expressing OSNs towards geosmin.

Besides signal reception in the dendritic compartment, signal transmission at the presynaptic AZ is an important site of modulation. Here, activity-dependent presynaptic plasticity can mediate short-term tuning and long-lasting changes of neuronal communication (Regehr, 2012; Monday et al., 2018). Our results describe fewer AZs in individual Or56a OSNs and a smaller total number of AZs belonging to Or56a-expressing neurons in DA2 glomeruli. However, the AZs of Or56a OSNs have higher levels of Unc13A than their counterparts in Or22a expressing neurons in DM2 glomeruli. Unc13 plays an essential role in synaptic vesicle priming at the AZ membrane (Varoqueaux et al., 2002). The *Drosophila* A isoform (Unc13A) positions vesicles close to voltage-gated calcium channels and thereby promotes neurotransmitter release. The B isoform (Unc13B) and the AZ protein Syd-1, on the other hand, are associated with loose coupling and less efficient neurotransmitter release (Böhme et al., 2016). Accordingly, strong expression of Unc13A and low levels of Unc13B and Syd-1 correlate with high release probability at peripheral and central AZs (Reddy-Alla et al., 2017; Fulterer et al., 2018). This molecular signature suggests reliable synaptic neurotransmitter release at AZs of Or56a OSNs. This feature would nicely match the high detection sensitivity of these neurons and reflect their physiological role in sensing and transmitting an alarm signal.

Homeostatic plasticity serves to maintain stable chemical synaptic transmission by counterbalancing disruptions. Homeostatic mechanisms stabilize sensory tuning features and several neurological and psychiatric diseases have been linked to dysregulated homeostatic synaptic plasticity (Wondolowski and Dickman, 2013; Davis and Müller, 2015; Frank et al., 2020; Wen and Turrigiano, 2024). Upon Cac knockdown, food odour-processing Or22a OSNs display such homeostatic regulation by increasing release site numbers within several days to compensate for a decrease in transmitter release probability. In contrast, we detected no compensatory addition of AZs in Or56a OSNs. Thus, these high-sensitivity neurons appear less plastic both at the dendritic level of signal detection (Halty-deLeon et al., 2024) and at the presynaptic site of signal transmission. These results point to an interesting similarity at the larval neuromuscular junction (NMJ). Here two glutamatergic motoneuron types possess AZs with low and high average release probabilities [type Ib and type Is, respectively; (Kurdyak et al., 1994; Lu et al., 2016)]. Intriguingly, at the NMJ long-term presynaptic homeostatic compensation also only operates at the low release probability motoneuron and matching our results for Or22a-expressing OSNs this plasticity involves an increase in Unc13A (Newman et al., 2017; Böhme et al., 2019; Chien et al., 2025). Given this heterogeneity, it will be of great interest to identify the molecular features that bestow synapses with particular plasticity properties and to elucidate how this differentiation is adapted to specific physiological and ethological demands.

## Data Availability

The original contributions presented in the study are included in the article/supplementary material, further inquiries can be directed to the corresponding authors.
